# Case Report: Diagnosis of splenic marginal zone lymphoma by ultrasound and contrast- enhanced ultrasound

**DOI:** 10.3389/fonc.2025.1569962

**Published:** 2025-08-20

**Authors:** Xu Zhang, Jianghao Lu, Yanli Hao, Tingting Wu, Zeyan Gao, Peng Zhou

**Affiliations:** ^1^ Department of Ultrasound, The Second People’s Hospital of Shenzhen (The First Affiliated Hospital of Shenzhen University), Shenzhen, Guangdong, China; ^2^ Department of Pathology, The Second People’s Hospital of Shenzhen (The First Affiliated Hospital of Shenzhen University), Shenzhen, Guangdong, China

**Keywords:** splenic marginal zone lymphoma, ultrasound, contrast-enhanced ultrasound, diagnostic analysis, primary splenic lymphoma

## Abstract

Early diagnosis of splenic marginal zone lymphoma (SMZL) is difficult because of its insidious symptoms and slow progression. Combining conventional ultrasound with contrast-enhanced ultrasound can enable to differentiate between benign and malignant splenic lesions, which could guide further clinical examination and eventually improve the diagnostic rate of SMZL.

## Introduction

Splenic marginal zone lymphoma (SMZL) is an extremely rare indolent B-cell lymphoma originating in the spleen. Its pathogenesis may involve complex multigene mutations induced by a diverse surrounding microenvironment (1). The WHO classifies SMZL as an independent lymphoid tissue tumor (2). SMZL is a low-grade malignant non-Hodgkin lymphoma (NHL) that accounts for approximately 0.6% of all NHL cases (3, 4). It occurs most frequently in the elderly population, with a median age of 68 years (5) and a median survival time of approximately 10 years; however, approximately 10% of patients show conversion of SMZL to diffuse large B-cell lymphoma (6). SMZL is often insidious with slow progression and a propensity for involvement of the spleen, bone marrow, and peripheral blood (7). In the early stage of the disease without bone marrow invasion, laboratory examination shows normal results and absence of apparent clinical symptoms; consequently, clinical detection of SMZL is difficult. Common clinical manifestations of SMZL are abdominal pain, back pain, splenomegaly, anemia, and weight loss (8). Splenomegaly is the most prominent clinical manifestation. Most patients show bone marrow invasion, while lymph nodes and extranodal involvement are rare. Histologically, SMZL may present as nodular lymphoid infiltration around the germinal center, with tumor cells originating from small lymphocytes.

Regarding imaging characteristics, because diffuse uniform tumor infiltration is the common pathological manifestation of SMZL, computed tomography (CT) and magnetic resonance imaging can only show spleen enlargement in SMZL, which is very difficult to differentiate from spleen enlargement due to non-neoplastic lesions and can be easily misdiagnosed as other diseases, thereby neglecting the possibility of lymphoma. Although a PET/CT scan can recognize the increased glucose metabolism of tumor cells and is superior to CT in differentiating spleen enlargement caused by SMZL and non-neoplastic lesions (9, 10), it is not used as a routine clinical examination because of its high cost and radiation side effects. Compared to these modalities, ultrasound has the following advantages: noninvasiveness, low cost, convenient operation, dynamic observation, and high repeatability. It is one of the first methods of clinical routine spleen examination and regular review; it can detect the size and shape of the spleen in an early stage, dynamically monitor the subtle changes in the splenic parenchymal echo, and determine the presence of space-occupying lesions in the spleen. Moreover, based on conventional ultrasound, ultrasound contrast examination can clearly show the blood perfusion pattern of spleen tissues and lesions and facilitate the differential diagnosis of spleen tumor lesions. According to previous literature, few studies have reported the use of ultrasonography and contrast examination to detect SMZL manifestations. Here, we report the case of a patient diagnosed to have SMZL through a combination of conventional ultrasound and contrast-enhanced ultrasound (CEUS). The enhancement pattern of the intrasplenic lesion in this case can serve as a reference for diagnosing splenic lymphoma by CEUS.

## Case description

The patient was a 65-year-old female. Although physical examination conducted 2 years ago showed spleen enlargement, the patient experienced no discomfort during rest and did not undergo further diagnosis and treatment for spleen enlargement. Recently, her body weight reduced by 2 kg, and she was admitted to the Shenzhen Second People’s Hospital for re-examination of spleen enlargement and further investigation of the underlying cause, in the absence of any apparent discomfort. Physical examination revealed the following findings: absence of mass in the entire abdomen; slight tenderness in the right upper abdomen; no rebound pain; no muscle tension; and no sensation under the liver, spleen, and ribs. Laboratory examination in our hospital showed the following results: reduced lymphocyte count (0.54 × 10^9^/L); lymphocyte ratio: 0.153; and normal range for red blood cell count, white blood cell count, platelet count, and hemoglobin level.

**Figure 1:**
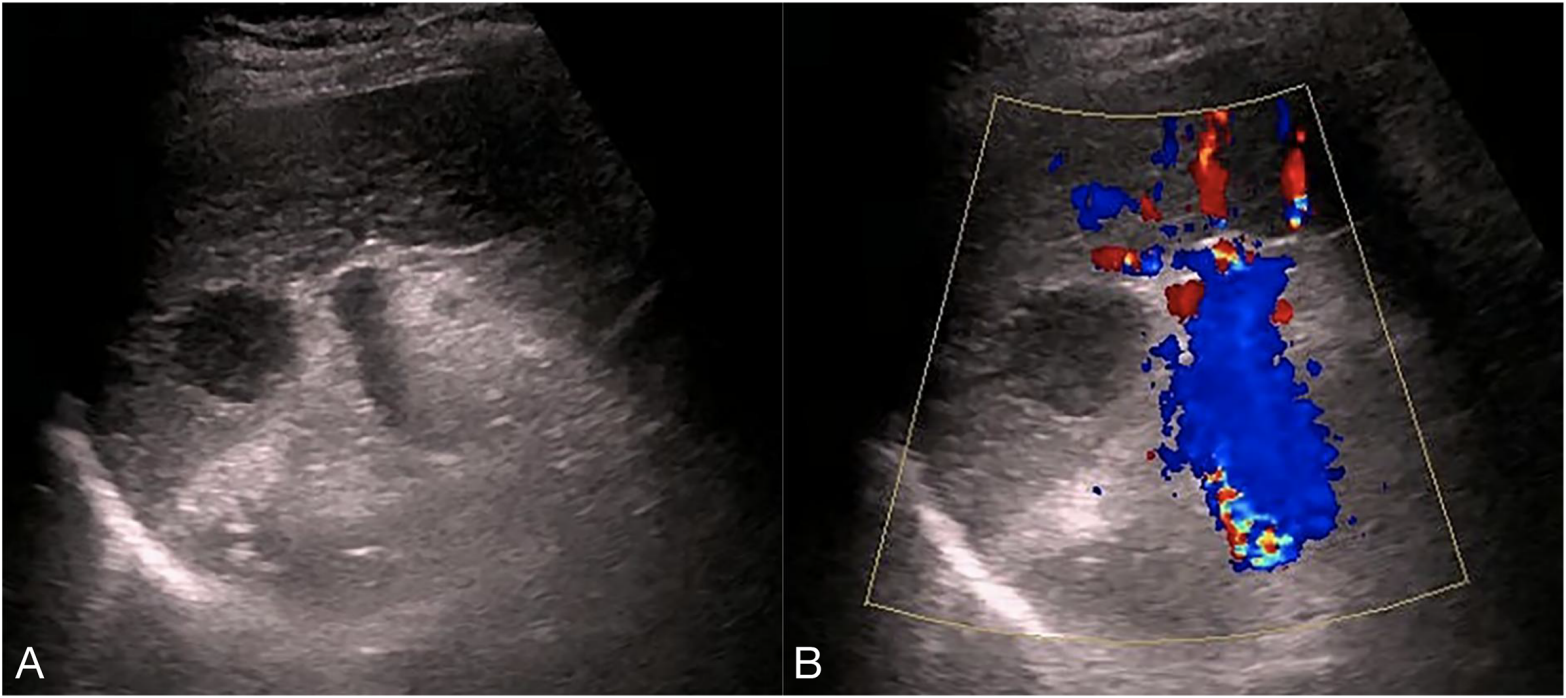
Two-dimensional ultrasound and Color Doppler flow images. **(A)** the spleen was enlarged, with approximately 52 mm thickness and 147 mm length, diffuse thickening and uneven distribution,of the parenchymal echo, and zonal- or grid-shaped. A mass was observed at the splenic hilum, with a size of approximately 24 × 22 mm, uneven surface, very low internal echo, oval shape, and clear boundary. **(B)** The mass at the splenic hilum showed a weak blood flow signal, with no apparent abnormality in the blood flow signal of the spleen.

**Figure 2:**
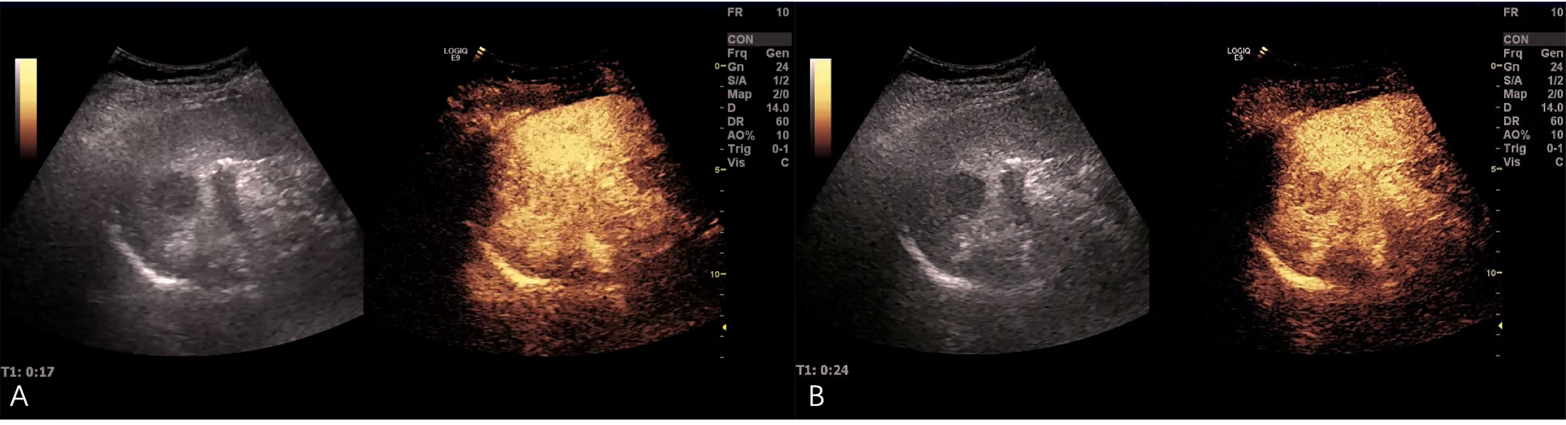
Contrast-enhanced ultrasound (CEUS) images. **(A)** After the infusion of the contrast agent SonoVue, the mass at the splenic hilum began to show inhomogeneous enhancement (low enhancement) in the arterial stage, and the enhanced edge was clear. The enhancement reached the peak at 17 s, and the peripheral splenic tissue showed uniform enhancement as compared to the mass. **(B)** The mass began to show a decline in enhancement at 24 s in the venous stage. The tumor mass showed delayed regression as compared to the surrounding tissue.

**Figure 3:**
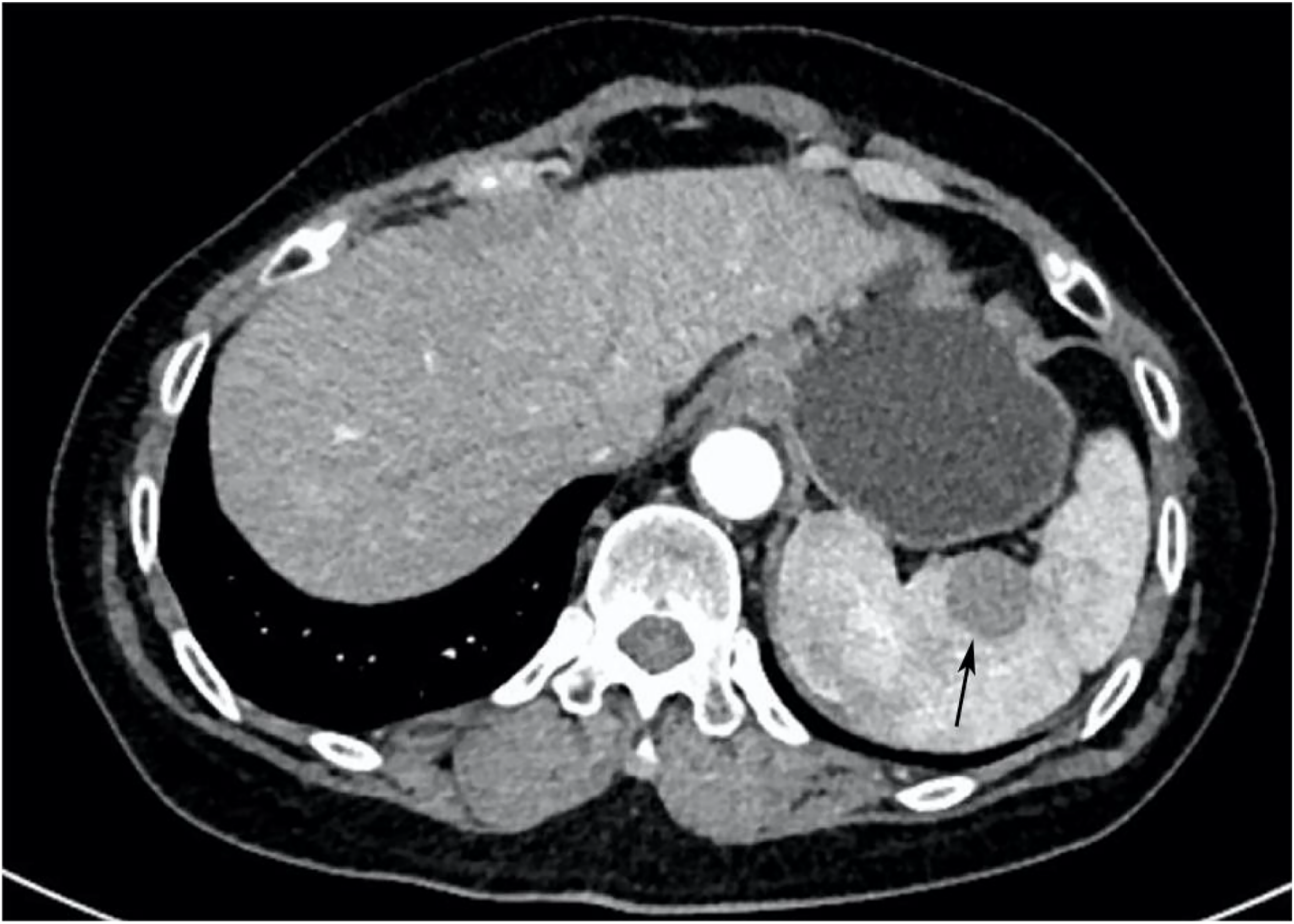
CT image. The spleen was enlarged and unevenly distributed; two isodense enhanced foci were observed in the spleen, with the larger one having approximately 20 mm diameter (indicated by the arrow).

Ultrasonography conducted 2 years ago showed an enlarged spleen with 42 mm thickness and 145 mm length. The splenic parenchyma exhibited a diffusely thickened echotexture with a reticular pattern and no space-occupying lesions were identified. Two-dimensional ultrasonography conducted in our hospital showed an enlarged spleen, with approximately 52 mm thickness and 147 mm length, diffuse thickening and uneven distribution of the splenic parenchymal echo, and zonal or grid-shaped. A mass of size approximately 24 × 22 mm was detected at the hilum of the spleen. It showed irregular surface, with a very low internal echo, oval shape, and clear boundary. Color Doppler flow imaging revealed a weak blood flow signal in the mass located at the splenic hilum, with no apparent abnormality in the blood flow signal in the spleen (**Figure 1**). To further qualitatively diagnose the mass located at the splenic hilum, CEUS was performed after receiving patient consent. Following the infusion of the contrast agent SonoVue, the mass located at the splenic hilum began to show inhomogeneous enhancement (low enhancement) in the arterial stage, with a clear enhanced edge. The enhancement reached the peak at 17 s, and the peripheral splenic tissue showed enhanced uniformity as compared to the mass. At the venous stage, the mass began to show a decline in enhancement at 24 s. Compared to the peripheral splenic tissue, regression of the tumor mass was delayed, and the overall enhancement pattern was slow-in and fast-out, as shown in **Figure 2**. Combined with the results of CEUS, the final diagnosis was splenomegaly with splenic lymphoma. Needle biopsy was recommended if necessary. CT imaging revealed an enlarged spleen with uneven distribution; two isodense enhanced foci were observed in the spleen, with the larger one showing a diameter of approximately 20 mm (**Figure 3**). Bone marrow aspiration showed the following pathological findings: CD20(+; a small number of B lymphocytes), CD3(−), CD138(−), and CD38(−). Bone marrow hyperplasia was slightly reduced.

## Differential assessment

The patient was asymptomatic with spleen enlargement detected during physical examination. During the review of spleen ultrasound findings, conventional ultrasound showed slow and continuous enlargement of the spleen, with grid-shaped, diffuse thickened echo of the splenic parenchyma. This finding was different from the commonly occurring splenomegaly caused by cirrhosis, congestive splenomegaly, chronic hemolytic diseases, excessive immune response, and infections, wherein the enlarged splenic parenchymal echo pattern is uniform and delicate. Additionally, in the present case, a strong hypoechoic mass was detected at the splenic hilum with a weak blood flow signal. The presence of an abnormal hypoechoic mass in the splenic hilum region indicates the possibility of splenic neoplastic lesions. The common diseases of splenic hypoechoic nodules represent several abnormal conditions, including splenic hemangioma, cysts containing thick fluid, splenic metastasis, and lymphoma. To further confirm whether the mass was benign or malignant, we conducted splenic CEUS. The mass located at the splenic hilum showed low inhomogeneous enhancement in the arterial phase and delayed regression in the venous phase, i.e., the “slow-in and fast-out” enhancement pattern. Thus, based on the findings that the benign splenic hypoechoic hemangioma showed slow peripheral hyperenhancement in the arterial stage and slow regression in the venous stage and the splenic thick-fluid cyst exhibited no blood perfusion in CEUS, the mass located at the splenic hilum in the present case was considered a malignant lesion. Although the CEUS findings for splenic metastases were similar to those observed in the present case, i.e., low enhancement in the arterial phase and rapid regression in the venous phase, conventional ultrasound showed that the mass was hypoechoic with a peripheral hypoechoic halo. Moreover, Dietrich (11) et al. demonstrated that high enhancement with rapid flushing on contrast-enhanced ultrasound is closely related to B-cell lymphoma (including SMZL), which can distinguish it from benign lesions, further confirming our diagnosis.

**Figure 4:**
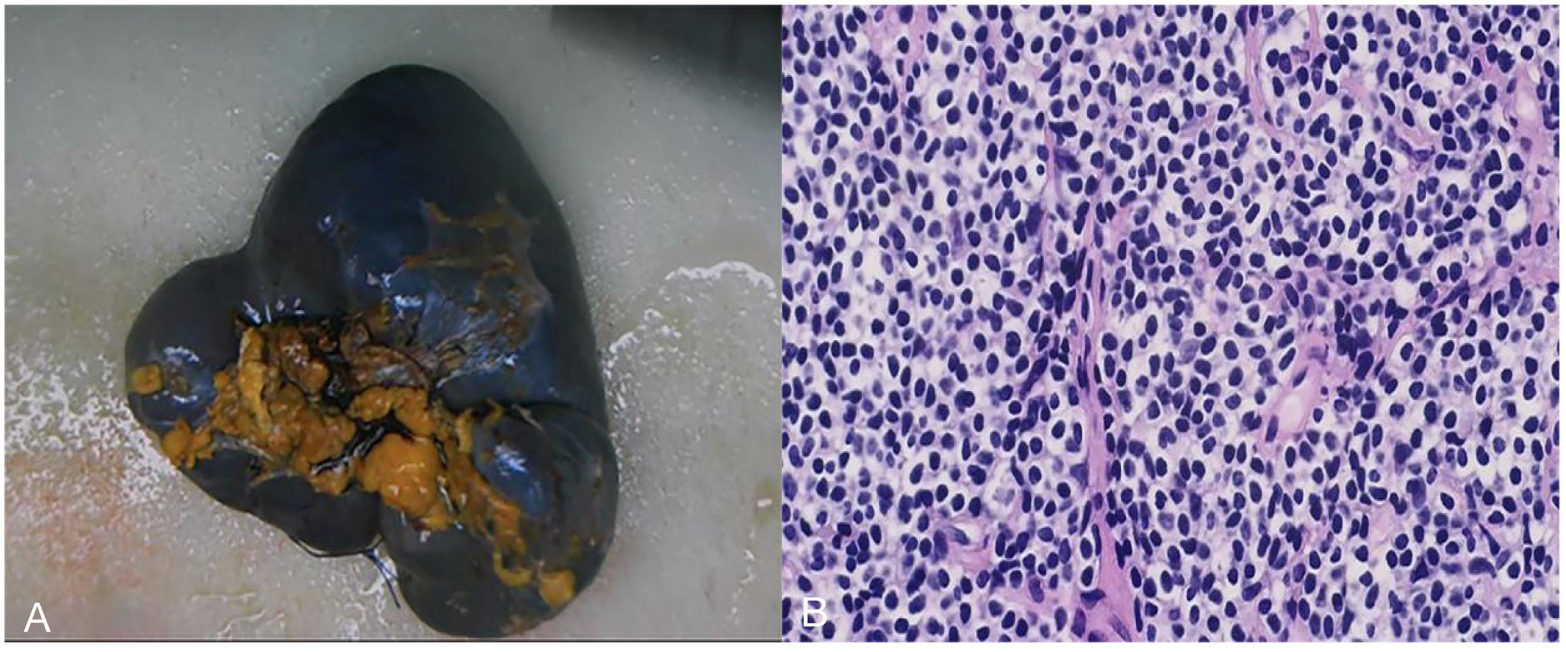
Postoperative gross observation and light microscopy images. **(A)** the spleen was enlarged and intact, and the spleen parenchyma was granular and nodular. A hard gray nodule with a clear boundary was observed in the spleen, with a size of approximately 2.5 × 2.0 × 2.2 cm. **(B)** Light microscopy image (200× magnification): diffuse proliferation of tumor cells, most of which were rich and bright mononuclear cells scattered among centroblasts and centrocytes.

The patient initially underwent splenectomy under general anesthesia and was discharged with good recovery. Postoperative gross observation showed that the spleen was enlarged and intact, and the splenic parenchyma was granular and nodular. The spleen also showed a hard gray nodule of approximately 2.5 × 2 × 2.2 cm in size, with a clear boundary. Light microscopy revealed diffuse proliferation of tumor cells, most of which were rich and bright mononuclear cells scattered among centroblasts and centrocytes (**Figure 4**). Immunohistochemical analysis showed the following findings: CD20(diffuse +), CD3(−), BCL2(diffuse +), BCL6(−), CD10(−), CD43(−), cyclinD1(−), CD21(−), CD23(−), and Ki67(+8%). Combined with pathological and immunohistochemical markers, the diagnosis of splenic marginal B-cell lymphoma was considered. The tumor invaded two lymph nodes located in the splenic hilum.

Following a one-month postoperative recovery period, four cycles of Rituximab-based chemotherapy were administered at monthly intervals. The patient underwent systematic postoperative imaging surveillance with alternating ultrasonography and computed tomography (CT) at scheduled intervals: 1, 2, 3, and 4 months, followed by annual evaluations at 1 and 2 years. No evidence of tumor recurrence or metastatic disease was detected throughout the follow-up period.

## Discussion

Primary SMZL is a rare tissue subtype of PSL in clinical settings (12) and is a slow-progressing, inert B-cell lymphoma originating from the spleen. Gross examination revealed proliferative expansion of the white pulp within the splenic parenchyma, forming multiple small to medium-sized grayish-white nodules. Histologically, the neoplastic cells exhibited a biphasic growth pattern within the splenic white pulp: Centrocyte-like cells were observed effacing the mantle zones, either peripherally surrounding or completely replacing the residual germinal centers. The red pulp demonstrated nodular infiltration by lymphocytes. In both bone marrow and lymph nodes, the neoplastic infiltration manifested as nodular interstitial infiltration, with cellular morphology morphologically congruent with that observed in the lymph nodes. The only typical manifestation of this lymphoma is enlargement of the spleen. In the absence of bone marrow invasion, the laboratory tests show normal results, without any apparent clinical symptoms. There are, however, many clinical causes of splenomegaly, such as hepatocirrhosis, congestive splenomegaly, chronic hemolytic diseases, excessive immune response, and infections. Therefore, the differential diagnosis of SMZL is rather difficult and requires a combination of clinical features and pathological examination after splenectomy for a confirmed diagnosis. In this case, SMZL can be differentiated from benign masses and metastases of the spleen based on ultrasound images and CEUS features. Based on the immunohistochemical analysis, SMZL cells express B cell-associated antigens (CD19, CD20, and CD22), surface immunoglobulins, and BCL2, but not CD5, CD10, CD23, CD11c, or CD43 (13) These characteristics can be used for the differential diagnosis of chronic lymphocytic leukemia, mantle cell lymphoma, and follicular lymphoma. The treatment of SMZL includes splenectomy, chemotherapy, and local radiotherapy of the spleen area (14, 15).

Ultrasound examination remains a widely used first-line imaging method for abdominal organ assessment in clinical research due to its convenience, real-time performance, cost-effectiveness and the absence of ionizing radiation, and its application is crucial to detect spleen enlargement in SMZL patients without clinical symptoms. In the present case, no hypoechoic halo was detected around the mass in conventional ultrasound, and the enlarged splenic parenchymal echo was diffusely thickened with a grid shape. Based on clinical manifestations, the possibility of splenic lymphoma is considered to be high in asymptomatic elderly people with slow enlargement of the spleen and the presence of an abnormal mass at the splenic hilum. The mass at the splenic hilum was eventually considered an invasive enlarged lymph node.

## Conclusions

SMZL is a rare disease, and its early diagnosis is difficult because of its insidious symptoms and slow progression. Conventional ultrasound and CEUS can improve the diagnostic rate of SMZL (16). In the present case, the findings of conventional ultrasound and CEUS combination guided the clinical diagnosis of SMZL. It is also imperative for clinicians to be aware of the clinical and pathological characteristics of SMZL during ultrasound diagnosis and to remain cautious regarding the patient’s symptoms for improving the early detection rate of this disease.

## Data Availability

The original contributions presented in the study are included in the article/supplementary material. Further inquiries can be directed to the corresponding author.
